# STING Activation in Macrophages and Microglia Drives Poststroke Inflammation: Implications for Neuroinflammatory Mechanisms and Therapeutic Interventions

**DOI:** 10.1111/cns.70106

**Published:** 2024-12-19

**Authors:** Zhiruo Liu, Qin Qin, Shisi Wang, Xinmei Kang, Yuxin Liu, Lei Wei, Zhengqi Lu, Wei Cai, Mengyan Hu

**Affiliations:** ^1^ Department of Neurology, Mental and Neurological Disease Research Center the Third Affiliated Hospital of Sun Yat‐sen University Guangzhou China; ^2^ Guangdong Provincial Key Laboratory of Brain Function and Disease Guangzhou China

**Keywords:** inflammation, macrophage, microglia, STING, stroke

## Abstract

**Background:**

Monocyte‐derived macrophages and microglia initially adopt an anti‐inflammatory phenotype following stroke but later transition to a pro‐inflammatory state. The mechanisms underlying this phenotypic shift remain unclear. This study investigates the activation dynamics of molecular signaling pathways in macrophages and microglia after stroke.

**Methods:**

We utilized publicly available single‐cell RNA sequencing datasets to examine the activation dynamics of molecular signaling pathways alongside the pro‐inflammatory phenotype of macrophages and microglia. Male C57BL/6 mice underwent transient middle cerebral artery occlusion (tMCAO), with the STING inhibitor H151 administered to tMCAO mice. Neurobehavioral performance was assessed using rotarod, foot fault, novel object recognition, and water maze tests at 5‐, 7‐, 10‐, and 14‐days post‐stroke. Primary microglia and bone marrow‐derived macrophages were cultured for in vitro experiments.

**Results:**

Single‐cell sequencing data indicated that the activation of STING and subsequent type I interferon signaling drove the phenotypic shift of microglia and macrophages toward a pro‐inflammatory state in the stroke lesion. Immunostaining demonstrated that the emergence of pro‐inflammatory microglia and macrophages aligned with the activation time course of STING and type I interferon signaling. Continuous phagocytosis by macrophages and microglia led to STING activation, which triggered type I interferon signaling and promoted the phenotypic shift. Inhibition of STING signaling prevented this transition, reduced neuroinflammation, and conferred protection against ischemic stroke.

**Conclusion:**

These findings elucidated the critical role of STING‐mediated type I interferon signaling in driving post‐stroke neuroinflammation and underscored the potential of STING inhibition as a therapeutic strategy for alleviating neuroinflammatory responses following stroke.

## Graphical abstract

After stroke, macrophages and microglia transition from an anti‐inflammatory to a proinflammatory phenotype due to lysosomal overload and impaired STING degradation. The activation of STING and the subsequent type I interferon signaling pathways results in the phenotypic transition of macrophages/microglia. Inhibiting STING can reverse the proinflammatory shift, thereby reducing poststroke neural inflammation and improving neurological outcomes.

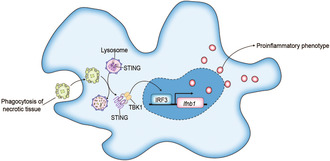

**Abbreviations**
BEAM: Branch expression analysis modeling
BMDMs: Bone marrow‐derived macrophages
CBF: Cerebral blood flow
cGAMP: Cyclic GMP‐AMP
cGAS: Cyclic GMP‐AMP synthase
DAMPs: Danger‐associated molecular patterns
ECA: External carotid artery
FACS: Flow cytometry
GAPDH: Glyceraldehyde‐3‐phosphate dehydrogenase
GO: Gene Ontology
GSEA: Gene set enrichment analysis
i.p.: Intraperitoneal injection
Iba1: Ionized calcium‐binding adaptor molecule 1
ICA: Internal carotid artery
IFN‐γ: Interferon γ
IL‐10: Interleukin‐10
IRFs: Interferon regulatory factors
JAK: Janus kinase
MAST: Model‐based analysis of single‐cell transcriptomics
MCA: Middle cerebral artery
MFI: Mean fluorescence intensity
MIHC: Multiplex immunohistochemistry
NF‐κB: Nuclear factor‐kappa B
NLRP3: NOD‐like receptor protein 3
PCA: Principal component analysis
pIRF3: Phospho‐interferon regulatory factor 3
PPAR‐γ: Peroxisome proliferator‐activated receptor gamma
PS: Penicillin–streptomycin
QC: Quality control
qd: Latin *Quaque die*
SEM: Standard error of the mean
Sham: Sham surgeries
STAT: Signal transducer and activator of transcription
STING: Stimulator of interferon genes
TLR: Toll‐like receptor
tMCAO: Transient middle cerebral artery occlusion
TNF‐α: Tumor necrosis factor alpha
UMAP: Uniform Manifold Approximation and Projection
WT: Wild type

## 1 Introduction

Ischemic stroke is a leading cause of mortality and disability worldwide. Poststroke neural inflammation plays a pivotal role in determining disease severity and prognosis. The immune responses within and around the stroke lesion involve the activation of various immune cells, including monocyte‐derived macrophages and microglia, as well as the release of inflammatory mediators. Understanding the mechanisms underlying poststroke neuroinflammation is crucial for developing targeted therapeutic interventions to mitigate brain injury and promote recovery.

Monocyte‐derived macrophages and microglia play crucial and dual roles in the context of stroke lesions, contributing to both tissue damage and repair processes. During the acute phase, these cells actively participate in debris clearance within the lesion and release anti‐inflammatory cytokines such as IL‐10, promoting inflammatory resolution. However, as the pathological process develops, microglia and macrophages in and around the infarct shift toward a proinflammatory phenotype, with downregulated efferocytic function and enhanced production of proinflammatory mediators. The precise mechanisms governing this phenotypic shift have remained elusive to date.

Recent research has generated considerable interest in exploring the involvement of STING (stimulator of interferon genes) and type I interferon signaling in shaping the proinflammatory characteristics of macrophages and microglia. It has been established that STING, a pivotal regulator of the innate immune response, plays a crucial role in activating type I interferon signaling. Upon activation, the STING pathway in macrophages and microglia has been shown to stimulate the production of proinflammatory cytokines and chemokines, thereby amplifying the proinflammatory properties of both cell types.

The conventional mode of STING activation occurs through the binding of the cyclic dinucleotide 2′3’‐cyclic GMP–AMP (cGAMP), which is produced by the DNA sensor cyclic GMP–AMP synthase (cGAS). Notably, an alternative cGAS‐ and cGAMP‐independent mechanism of STING activation has also been identified [1]. Under normal conditions, STING is targeted for degradation in lysosomes; however, it may evade this pathway when lysosomes are impaired or excessively occupied. In the context of stroke, microglia and macrophages are heavily engaged in efferocytic activities. The potential escape of STING from lysosome‐dependent degradation and its role in driving the phenotypic shift of microglia and macrophages in this context warrant further investigation.

In this study, we demonstrate a concurrent activation pattern between type I interferon signaling and the proinflammatory phenotype of macrophages/microglia, as revealed through analysis of public datasets of single‐cell RNA sequencing and subsequent experimental validations. We identified that continuous phagocytosis by macrophages and microglia led to the activation of STING, thereby triggering type I interferon signaling and driving the phenotypic shift toward a proinflammatory state. Notably, inhibition of STING activity reversed the heightened proinflammatory properties observed in macrophages and microglia undergoing prolonged phagocytosis, consequently mitigating poststroke neural inflammation and improving neurological deficits. Our findings highlight the potential therapeutic significance of targeting STING activity to alleviate enhanced proinflammatory properties in macrophages and microglia, ultimately offering a promising strategy for ameliorating poststroke neural inflammation and improving neurological deficits.

## 2 Results

### 2.1 Activation Dynamic of Type I Interferon Signaling Coincides With the Phenotypic Shift of Macrophages/Microglia Toward Proinflammatory Subset

**FIGURE 1:**
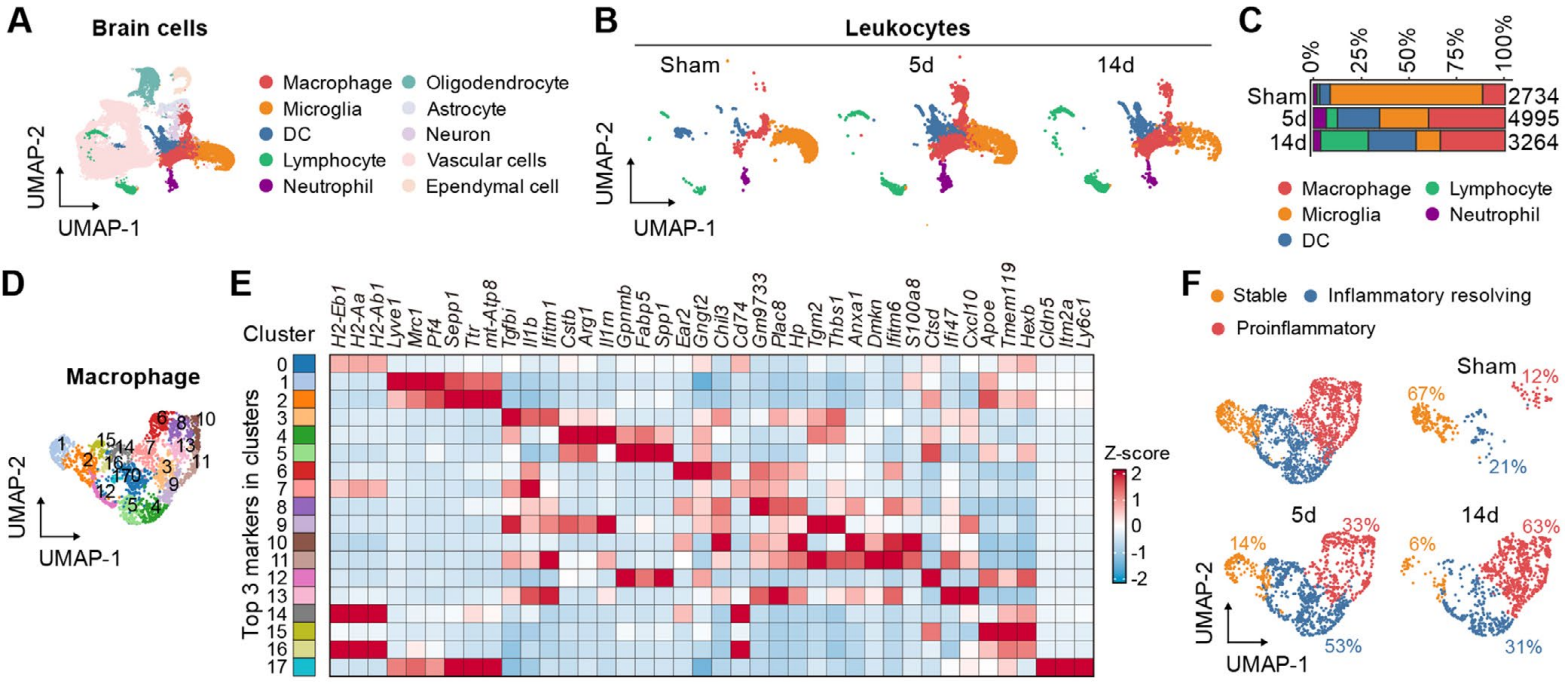
Phenotype shift in macrophages during stroke progression. Single‐cell sequencing data were integrated from sham surgeries (Sham, GSE227651) 5 and 14 days poststroke (tMCAO5d and tMCAO14d, GSE171171). Low‐quality cells and doublets were filtered out from each dataset. (A) Identification of 10 cell clusters by unsupervised clustering and Uniform Manifold Approximation and Projection (UMAP) based on established marker genes and cell type annotations. Changes in the relative abundance of each cell type over the stroke timeline. (B) Proportions of different cell populations poststroke. (C) Percentages of different cell subpopulations at various poststroke time points. (D) UMAP division of macrophages into 18 distinct subclusters. (E) Top three marker genes for each macrophage cluster and average expression of classic inflammatory related genes. (F) Identification of three main macrophage subtypes during stroke progression.

To investigate the phenotypic transition of macrophages and microglia following acute ischemic stroke, we integrated single‐cell sequencing data from sham surgeries (Sham) and from 5 and 14 days poststroke (tMCAO5d and tMCAO14d). After filtering out low‐quality cells and doublets, we performed unsupervised clustering and Uniform Manifold Approximation and Projection (UMAP), identifying 10 distinct primary cell clusters among 47,611 cells. Using established marker genes and unsupervised cell type annotations, we classified these clusters into macrophages, microglia, DC cells, lymphocytes, neutrophils, oligodendrocytes, astrocytes, neurons, vascular cells, and ependymal cells (Figure 1A and Figure S1A). By analyzing CD45^+^ immune cells during stroke progression, we observed significant changes in the relative abundance of each cell type. Consistent with previous reports [2, 3], macrophages and microglia increased at tMCAO5d, while lymphocytes significantly increased at tMCAO14d (Figure 1B,C). To determine the transcriptional profile changes in macrophages over the course of stroke, we reclustered the macrophage population, resulting in 18 distinct clusters (Figure 1D). Based on the highest average expression of the top three marker genes in each cluster and classic proinflammatory and anti‐inflammatory genes [4], we categorized macrophages into three main subtypes: stable phenotype, inflammation resolving phenotype, and proinflammatory phenotype (Figure 1E,F). As anticipated, the inflammation resolving phenotype increased at tMCAO5d, whereas the proinflammatory phenotype showed a more pronounced increase at tMCAO14d. Similar patterns were observed in microglia (Figure S2A–C).

**FIGURE 2:**
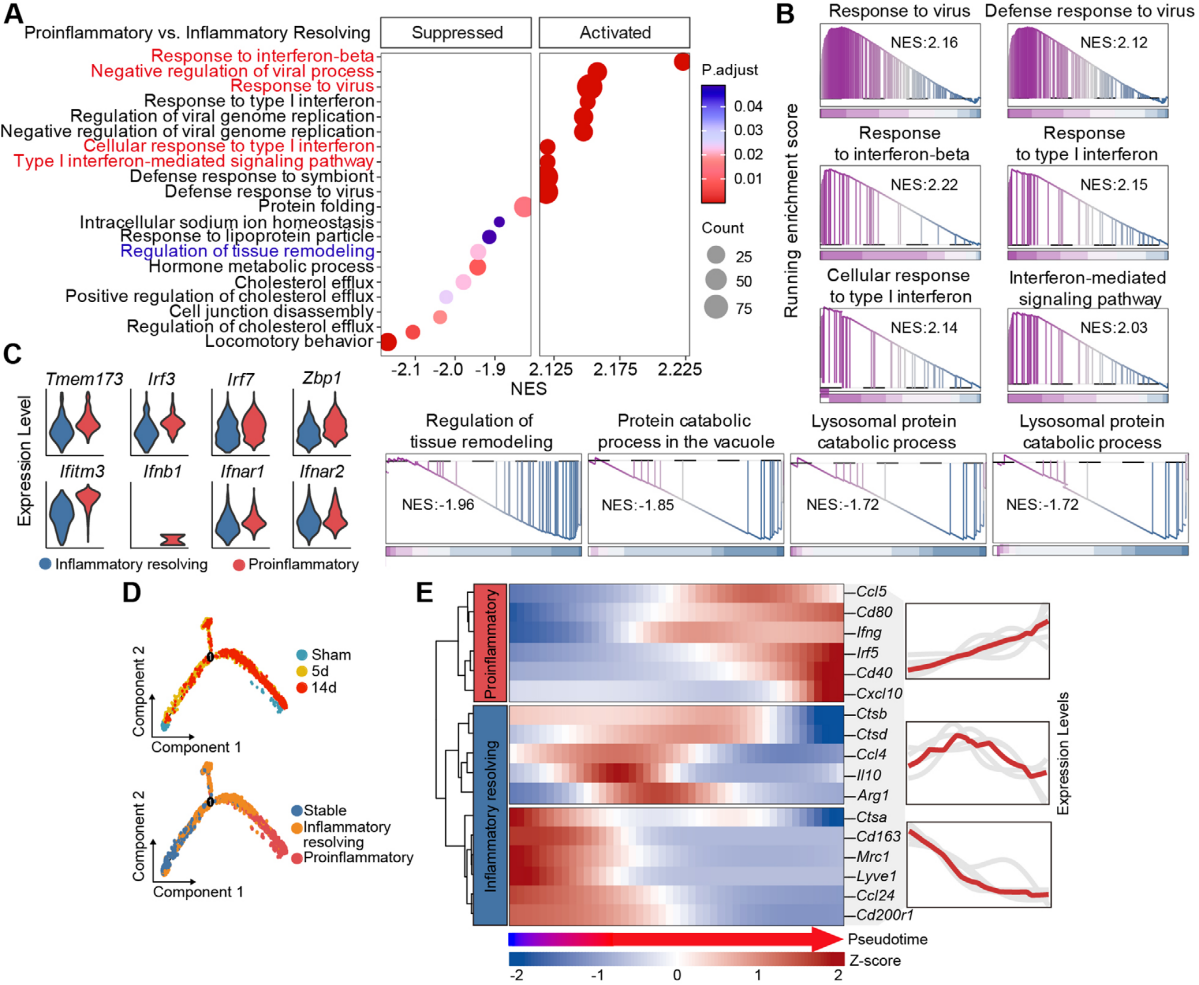
Activation of STING (stimulator of interferon genes) and type I interferon signaling in macrophage phenotypic shift. Single‐cell sequencing data were integrated from sham surgeries (Sham, GSE227651) 5 and 14 days poststroke (tMCAO5d and tMCAO14d, GSE171171). Low‐quality cells and doublets were filtered out from each dataset. Gene set enrichment analysis (GSEA) of differentially expressed genes in proinflammatory macrophages. (A) Activated and suppressed biological processes were presented. (B) Running enrichment scores for the listed biological processes. (C) Expression patterns of STING pathway‐related genes in different macrophage phenotypes. (D) Pseudotime trajectory of macrophages throughout the stroke timeline predicting three macrophage states. (E) Coordinated expression analysis of classical anti‐inflammatory and proinflammatory genes along the pseudotime trajectory.

To further investigate the functional transformation of macrophages and microglia after stroke, we first conducted Gene Set Enrichment Analysis (GSEA) between the proinflammatory phenotype and inflammation resolving phenotype of macrophages. The results indicated a significant upregulation of biological processes related to viral defense and type I interferon pathway activation, along with downregulation of the regulation of tissue remodeling (Figure 2A,B). Simultaneously, GSEA between tMCAO14d and 5d macrophages showed significant enrichment in type I interferon‐related biological processes (Figure S1B,C). STING and its downstream signaling molecules jointly regulate the expression of type I interferon [5]. We found that STING pathway‐related genes were significantly upregulated in the proinflammatory phenotype of macrophages compared to the inflammatory resolving phenotype (Figure 2C). Pseudotime trajectory predicted the progression of Sham macrophages toward tMCAO5d macrophages and subsequently toward tMCAO14d macrophages. In this trajectory, macrophages exhibit three states: initially a stable phenotype, transitioning to a resolving phenotype at the midpoint, and finally adopting a proinflammatory phenotype (Figure 2D). We further analyzed the coordinated expression patterns of classical proinflammatory and anti‐inflammatory genes along the pseudotime trajectory. As anticipated, the inflammatory resolving genes exhibited high coordinated expression during the early to mid‐stages, whereas the proinflammatory gene modules demonstrated high coordinated expression during the late stages (Figure 2E). Similar patterns were observed in microglia (Figure S2C–G). Therefore, we deduce that the activation of STING and the subsequent type I interferon signaling propel the phenotypic shift of microglia and macrophages toward a proinflammatory phenotype in the stroke lesion.

**FIGURE 3:**
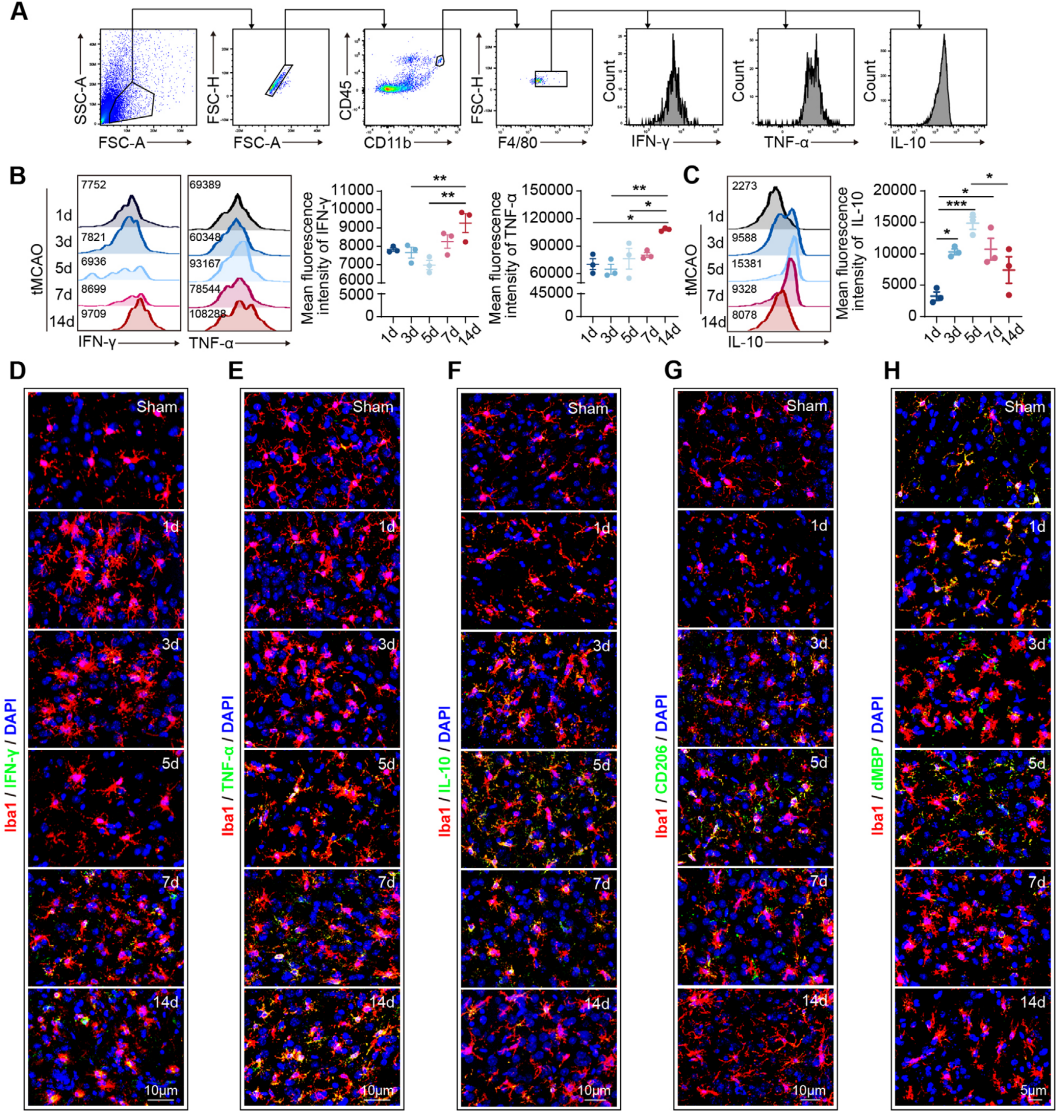
The phenotypic dynamics of microglia and macrophages in the stroke‐affected brain. Male C57/BL6J mice (aged 8–12 weeks) were subjected to 60 min of transient middle cerebral artery occlusion (tMCAO), and brain tissue was collected for flow cytometric analysis (A–C) and immunostaining (D–H) at specified time points. (A) Representative dot plots showing the gating strategy for macrophages. (B) Mean fluorescence intensity (MFI) of interferon γ/tumor necrosis factor α (IFN‐γ/TNF‐α) in macrophages from 1 to 14 days poststroke. *N* = 3 per group, *p < 0.05, **p < 0.01; by one‐way ANOVA. (C) Mean fluorescence intensity (MFI) of interleukin‐10 (IL‐10) in macrophages from 1 to 14 days poststroke. *N* = 3 per group, *p < 0.05, ***p < 0.001; by one‐way ANOVA. (D–H) Representative images of coronal brain slices collected at specific time points post‐tMCAO showing the nuclear (DAPI, blue) localization of Iba1^+^ (red) cells and IFN‐γ/TNF‐α/IL‐10/CD206/dMBP (green).

To test this hypothesis, we initially evaluated the phenotypic dynamics of microglia and macrophages in the stroke‐affected brain. Male C57/BL6J mice (aged = 8–12 weeks) were subjected to 60 min of transient middle cerebral artery occlusion (tMCAO), and brain tissue was collected for analysis at specified time points. Previous studies indicate that macrophages/microglia in the ischemic area begin expressing inflammatory factors 1 to 3 days after stroke, with levels gradually increasing until day 14. Concurrently, these macrophages/microglia also start to express anti‐inflammatory factors within the same time frame, peaking on day 5, decreasing by day 7, and returning to baseline levels by day 14 [6, 7, 8]. Through flow cytometric analysis (Figure 3A–C) and immunostaining (Figure 3D–G), we demonstrated that within 14 days postischemic stroke, the proinflammatory characteristics of microglia and macrophages gradually accumulated, as evidenced by the progressively elevated expression of IFN‐γ and TNF‐α (Figure 3B,D,E). Conversely, the expression of anti‐inflammatory mediators, including IL‐10 and CD206, increased at 1–5 days post‐tMCAO, but decreased from 7 days poststroke (Figure 3C,F,G). Concurrently, we observed a gradual decline in the phagocytic capacity of microglia and macrophages (Figure 3H). These findings are in line with previous reports [6, 9] and indicate that day 7 marks a pivotal shift in the phenotype of microglia and macrophages from an anti‐inflammatory to a proinflammatory subset.

**FIGURE 4:**
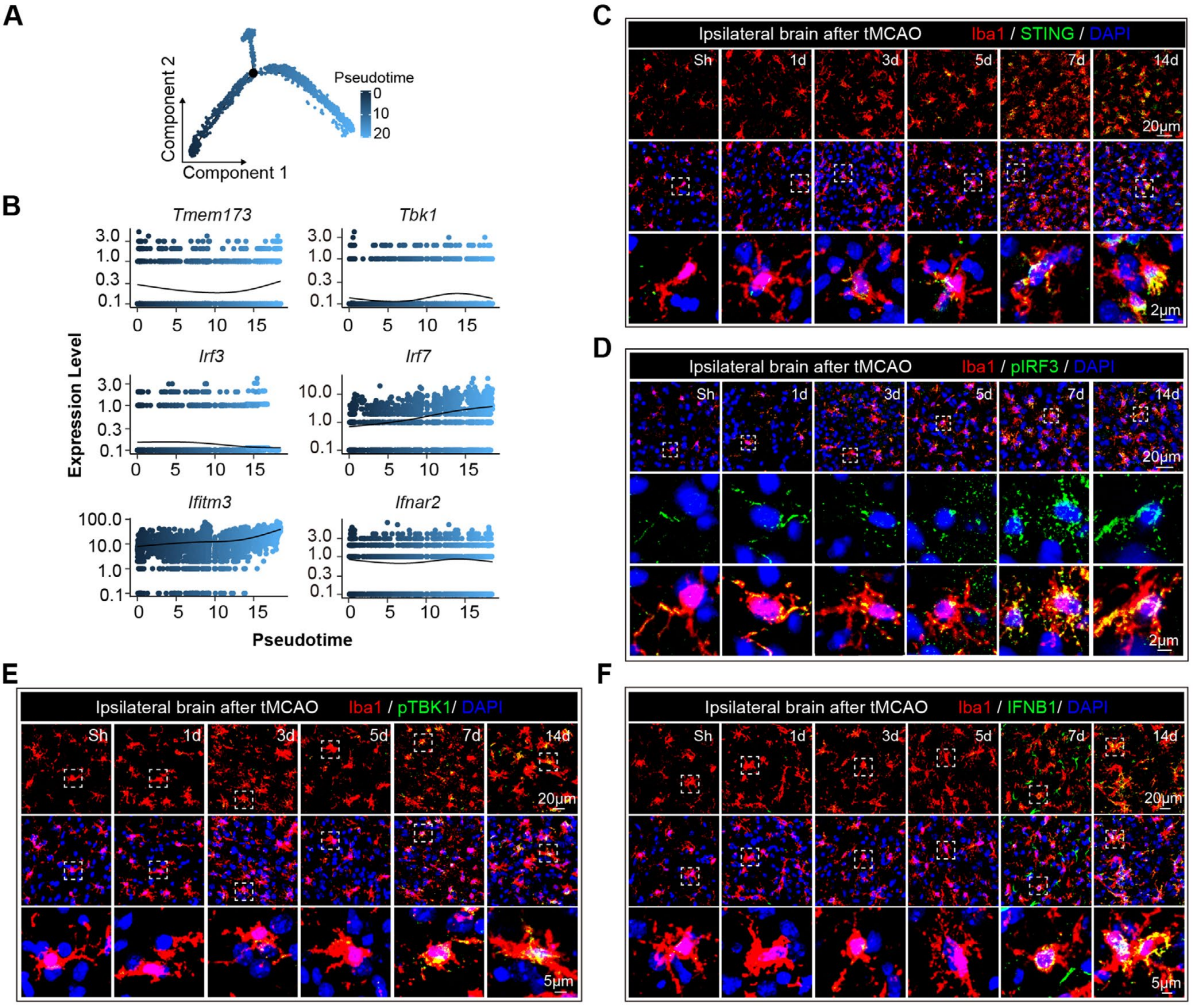
The activation dynamics of STING (stimulator of interferon genes) and type I interferon signaling in microglia and macrophages poststroke. Single‐cell sequencing data were integrated from sham surgeries (Sham, GSE227651) 5 and 14 days poststroke (tMCAO5d and tMCAO14d, GSE171171). Low‐quality cells and doublets were filtered out from each dataset. Pseudotime analysis of single‐cell sequencing data. (A) Pseudotime trajectory of STING activation throughout the stroke timeline. (B) The expression levels of downstream signaling molecules of STING. (C–F) Male C57/BL6J mice (aged 8–12 weeks) were subjected to 60 min of tMCAO, and brain tissue was collected for immunostaining at specified time points. Representative images of ipsilateral brain tissue collected from 1 to 14 days poststroke, showing the nuclear (DAPI, blue) localization of Iba1^+^ (red) cells and STING/pIRF3/pTBK1/IFNB1 (green). Dashed lines highlight the colocalization of STING/pIRF3/pTBK1/IFNB1 with the nuclei in Iba1^+^ cells.

In the pseudotime analysis of single‐cell sequencing data, we observed a progressive activation trend of STING and its downstream signaling molecules (Figure 4A,B and Figure S2H,I). To further explore these findings, we examined the activation dynamics of STING and type I interferon signaling. Immunostaining revealed an incremental increase in the expression of STING (Figure 4C and Figure S3A), pIRF3 (Figure 4D and Figure S3A), pTBK1 (Figure 4E), and IFNB1 (Figure 4F) from 1 to 14 days post‐tMCAO. Notably, from 7 days poststroke, Iba1^+^ macrophages and microglia exhibited significant STING, pIRF3, pTBK1, and IFNB1 expression (Figure 4C–F and Figure S3A). These data suggest that the development of proinflammatory microglia and macrophages coincided with the activation time course of STING and type I interferon signaling.

### 2.2 Prolonged Phagocytosis Facilitates STING Activation and Enhances Proinflammatory Properties of Microglia and Macrophages

It has been established that the degradation of STING is contingent upon the normal function of lysosomes [10]. Inhibition of lysosomal degradation of STING primes its activation and subsequently triggers the type I interferon signaling pathway [11]. Within the lesion of acute ischemic stroke, macrophages and microglia undertake the task of efferocytosis, during which lysosomes may become overloaded with phagocytic cargo. We therefore postulate that the overloading of lysosomes in microglia and macrophages facilitates the escape of STING, leading to the activation of the type I interferon signaling pathway and the augmentation of their proinflammatory properties.

**FIGURE 5:**
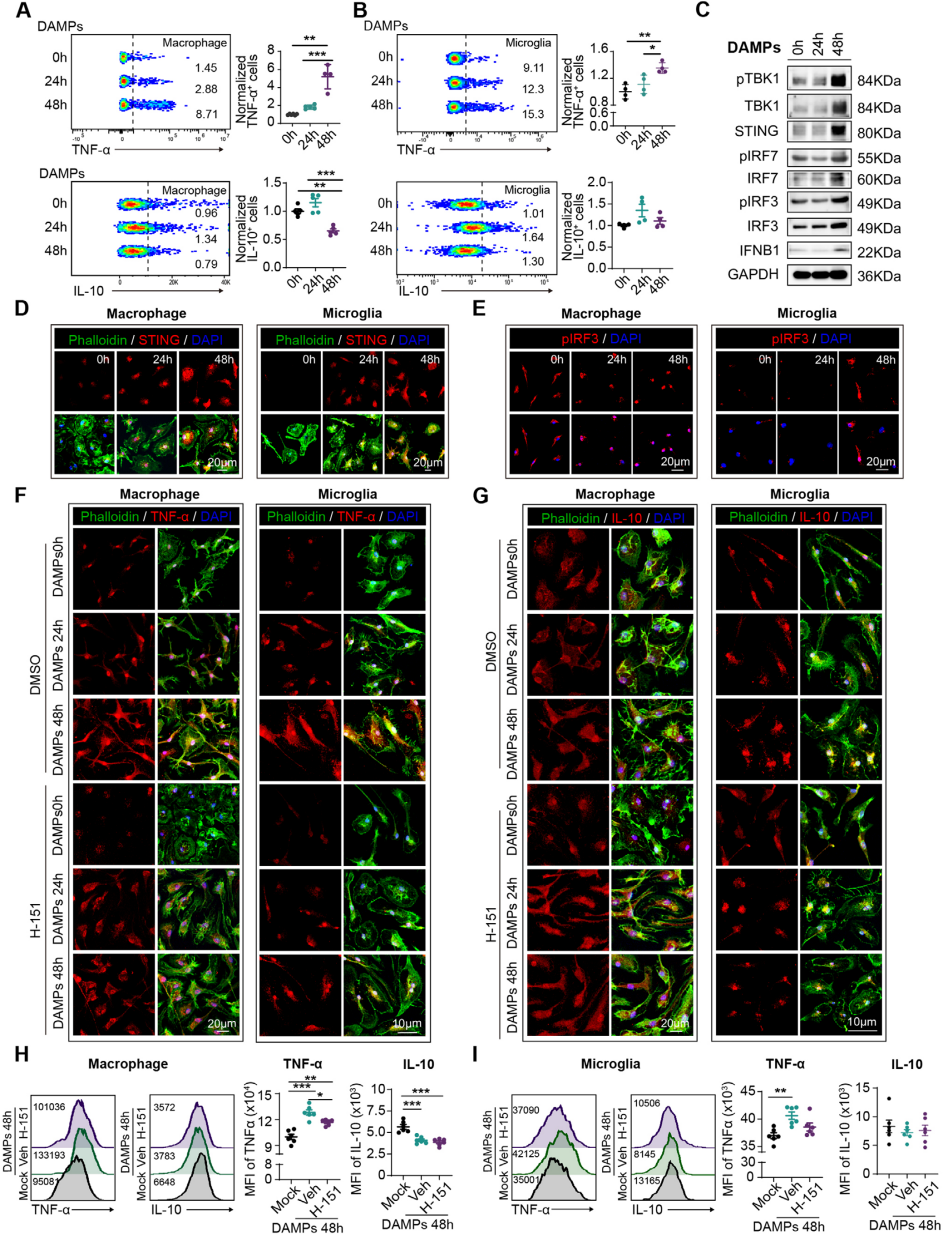
Prolonged phagocytosis facilitates STING (stimulator of interferon genes) activation and phenotypic shift in microglia and macrophages. A mixture of brain‐derived danger‐associated molecular patterns (DAMPs) was extracted from naïve mouse brains and administered to primary macrophage and microglia for short‐term (24 h) and prolonged (48 h) phagocytosis. Following treatment, we assessed the inflammatory properties and STING activation in microglia and macrophages. Representative plots and the percentage of TNF‐α^+^/IL‐10^+^ macrophages (A) and microglia (B) in control, short‐term, and prolonged phagocytosis groups. Experiments were conducted four times. *p < 0.05, **p < 0.01, ***p < 0.001 versus control; by one‐way ANOVA. (C) Western blot analysis revealed activation of the STING and type I interferon pathways in macrophages subjected to prolonged DAMP phagocytosis. Experiments were conducted three times, with statistical analysis presented in Figure S3B. (D, E) Immunostaining images indicating the localization of phalloidin (green), STING/pIRF3(red)and DAPI (blue) in macrophages and microglia. Experiments were conducted four times, with statistical analysis presented in Figure S3C. (F–I) The specific inhibitor of STING, H‐151(1 μmol/L), was applied to microglia and macrophages for 48 h. The expression of TNF‐α and IL‐10 was evaluated by immunostaining (F, G) and flow cytometric analysis (H, I). (F, G) Representative images showing the localization of phalloidin (green), TNF‐α/IL‐10 (red), and DAPI (blue) in macrophages and microglia after H‐151 treatment. Mean fluorescence intensity (MFI) of TNF‐α/IL‐10 in macrophages (H) and microglia (I). Experiments were conducted six times. *p < 0.05, **p < 0.01, ***p < 0.001; by one‐way ANOVA.

To experimentally validate this hypothesis, we prepared cultures of bone marrow‐derived macrophages (BMDMs). A blend of brain‐derived danger‐associated molecular patterns (DAMPs) was extracted from naïve mouse brains using the previously described methodology [12] and administered to the BMDMs for phagocytosis. Through flow cytometric analysis, we observed that prolonged phagocytosis of DAMPs (48 h) by macrophages (Figure 5A) and microglia (Figure 5B) induced a remarkable increase in TNF‐α expression compared to those subjected to short‐term phagocytosis (24 h) and the mock control (0 h). Conversely, the level of the anti‐inflammatory marker IL‐10 increased in macrophages after long‐term phagocytosis (Figure 5A), while it remained stable in microglia at the two tested time points (Figure 5B).

Subsequently, we evaluated the activation of STING and the type I interferon signaling pathway in macrophages (Figure 5C). Western blot analysis revealed robust activation of the STING and type I interferon pathway in the prolonged DAMPs phagocytosis groups compared to the controls (24 h and 0 h) (Figure 5C and Figure S3B). Immunostaining further demonstrated the expression of STING and the intranuclear translocation of pIRF3 in both macrophages and microglia (Figure 5D,E and Figure S3C) at 48 h after DAMPs treatment, providing additional support for the notion that the overloading of lysosomes in macrophages and microglia dictates the escape of STING, subsequent activation of the type I interferon signaling pathway, and the enhancement of their proinflammatory properties.

### 2.3 Inhibition of STING Signaling Interrupts the Phenotypic Shift of Microglia and Macrophages Toward Proinflammatory Phenotype After Stroke

We then investigated whether inhibiting STING activation could disrupt the phenotypic shift of macrophages and microglia toward proinflammatory phenotype after prolonged phagocytosis. Through immunostaining (Figure 5F,G) and flow cytometric analysis (Figure 5H,I), we demonstrated that the application of the specific STING inhibitor H‐151 reversed the substantial increase in TNF‐α levels at 48 h after DAMPs phagocytosis in both macrophages and microglia. Meanwhile, the expression of IL‐10 appeared to remain unaffected by H‐151 treatment (Figure 5F–I). Additionally, we found that the phagocytic function of microglia and macrophages, which declined after prolonged phagocytosis, could be reversed by treatment with H‐151 (Figure S4A,B).

**FIGURE 6:**
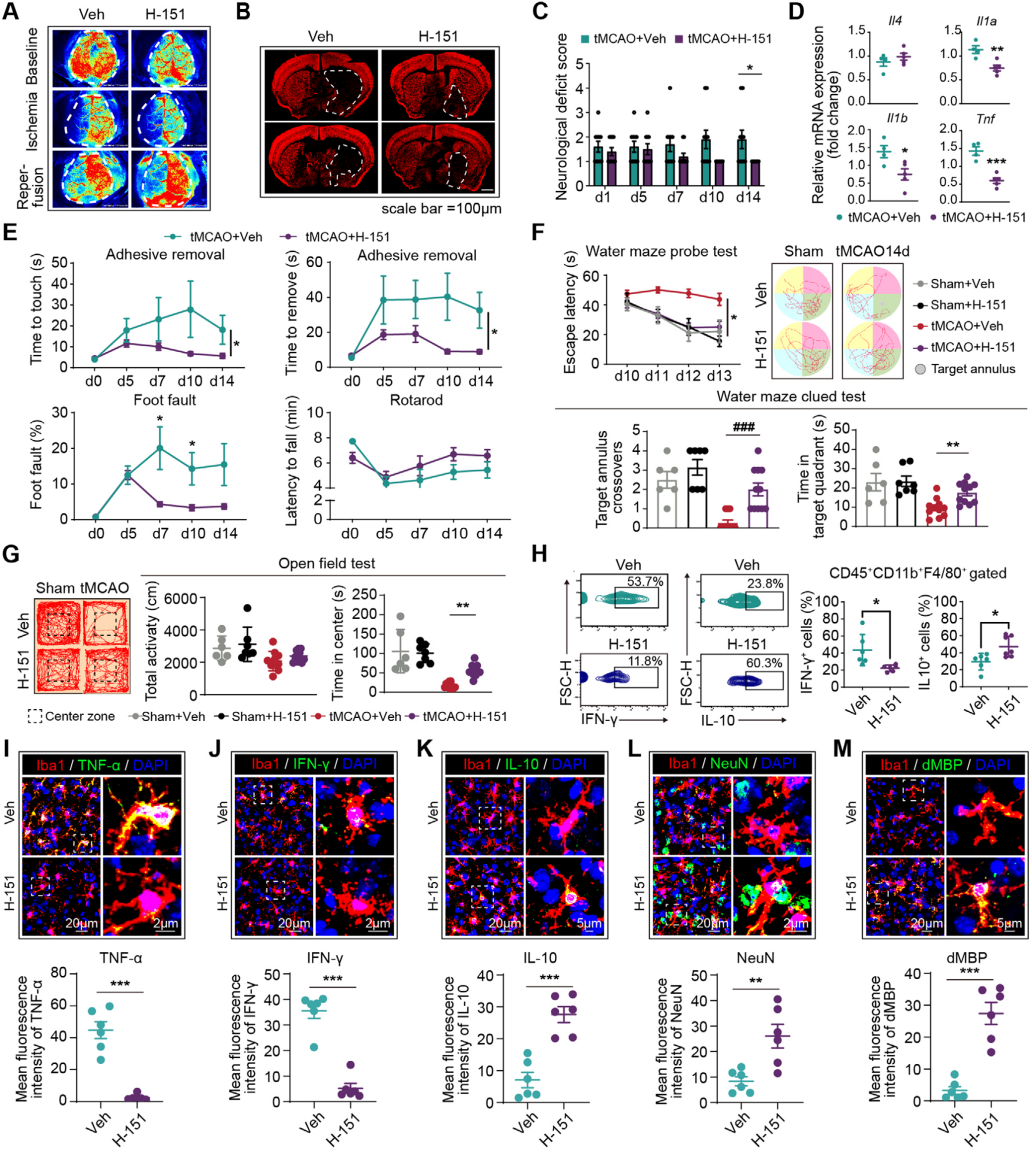
H‐151 inhibits the phenotype shift of microglia and macrophages, improving the stroke condition. Male C57/BL6J mice (aged 8–12 weeks) were subjected to 60 min of transient middle cerebral artery occlusion (tMCAO). H‐151 (10 mg/kg) was administered to mice from days 5 to 14 after tMCAO via intraperitoneal injection (i.p.), once daily (qd). The severity of the stroke condition was assessed. (A) Cerebral blood flow (CBF) comparison between control and H‐151 treated mice. *N* = 10 in each group. Statistical analysis presented in Figure S5B. (B) Representative images of infarct volume in tMCAO mice 14 days poststroke. Scale bar = 1 mm. Dashed lines outline the infarct area. *N* = 10 in each group. Statistical analysis presented in Figure S5C. (C) Neurological deficit scores assessed from days 1 to 14 post‐tMCAO. *N* = 10 in each group. *p < 0.05; by the Wilcoxon rank‐sum test. (D) PCR analysis revealed the expression of inflammation‐related mRNA in control and H‐151 treated mice poststroke. *N* = 5 in each group, *p < 0.05; by Student's *t* test. (E) Sensorimotor deficits evaluated using adhesive removal, rotarod, and foot fault tests from 5 to 14 days post‐tMCAO. *N* = 10 in each group. *p < 0.05; by two‐way ANOVA. Cognitive function assessed using the Morris water maze (F) and open field test (G) from days 10 to 13 post‐tMCAO. (F) Mice were subjected to four serial probe tests (1 test per day, starting from 10 days after tMCAO) and a clued test (14 days after tMCAO) of Morris water maze. In the probe tests. *N* = 6 in Sham group and *N* = 10 in tMCAO group. *p < 0.05; by two‐way *ANOVA*. In the clued test, ^###^ *p* < 0.001; by rank‐sum test. **p < 0.05; by one‐way *ANOVA*. (G) Mice were subjected to open field test on day 10 post‐tMCAO. *N* = 6 in Sham group and *N* = 10 in tMCAO group. **p < 0.05; by one‐way *ANOVA*. (H) Percentage of IFN‐γ^+^/IL‐10^+^ cells in CD45^+^CD11B^+^F4/80^+^ cells in vehicle (Veh) and H‐151 treated tMCAO mice. *N* = 6 in each group. *p < 0.05; by Student's *t* test. (I–M) Representative images of coronal brain slices collected at day 14 poststroke showing the nuclear (DAPI, blue) localization of Iba1^+^ (red) cells and IL‐10/TNF‐α/IFN‐γ/NeuN/dMBP (green) in Veh and H‐151 treated groups. Dashed lines outline the colocalization of IL‐10/TNF‐α/IFN‐γ/NeuN/dMBP with the nuclei of Iba1^+^ cells. **p < 0.01, ***p < 0.001; by Student's *t* test.

Subsequently, we assessed the therapeutic efficacy of H‐151 in stroke models and its impact on the phenotypic shift of macrophages and microglia. Previous studies have shown that following intraperitoneal administration, H‐151 achieves effective systemic levels, including in the central nervous system, and exhibits a short serum half‐life [13, 14]. Treatment with H‐151 inhibited the activation of the cGAS‐STING pathway in the brain, as evidenced by decreased levels of p‐TBK1, p‐p65, and p‐IRF3 proteins [15]. Given that day 5 marks the turning point in the phenotype of microglia and macrophages, H‐151 was administered to mice from this time point (days 5–14, i.p., 10 mg/kg, qd) (Figure S5A). Our findings demonstrated that delayed H‐151 treatment still led to improved disease outcomes. The tMCAO mice treated with H‐151 exhibited reduced infarct volume (Figure 6A,B and Figure S5B,C), ameliorated neurological deficits (Figure 6C), and enhanced neurological function (Figure 6E–G and Figure S5D). Correspondingly, PCR analysis revealed a reduction in neural inflammation in the H‐151 treated mice (Figure 6D). Through flow cytometric analysis (Figure 6H) and immunostaining (Figure 6I–M), we observed that H‐151 treatment downregulated the expression of the proinflammatory cytokine TNF‐α and IFN‐γ in macrophages and microglia (Figure 6I,J), while increasing the expression of IL‐10 (Figure 6K) and phagocytic capacity (Figure 6L,M). These data indicate that the suppression of STING signaling inhibits the phenotypic shift of microglia and macrophages toward a proinflammatory phenotype, subsequently ameliorating neural inflammation and providing protection against ischemic stroke.

## 3 Discussion

This study investigated the phenotypic transition of microglia and monocyte‐derived macrophages following stroke and elucidated the underlying molecular mechanisms. The research uncovered a simultaneous activation of type I interferon signaling and the proinflammatory phenotype of microglia and macrophages. We validated these findings and demonstrated that continuous phagocytosis triggers STING activation, leading to type I interferon signaling and driving the phenotypic shift toward a proinflammatory state. Notably, inhibiting STING activity reversed the heightened proinflammatory properties of microglia and macrophages following prolonged phagocytosis, subsequently ameliorating poststroke neural inflammation and neurological deficits. These findings have the potential to inform the development of targeted therapies aimed at modulating neuroinflammatory responses following stroke, with broader implications for the treatment of neuroinflammatory conditions. The study's use of publicly available datasets and experimental validation also underscores the translational relevance of the findings, offering a comprehensive approach to understanding and potentially addressing poststroke neuroinflammation.

Transcription factors play a crucial role in controlling the phenotype of microglia and macrophages which include nuclear factor‐kappa B (NF‐κB), signal transducer and activator of transcription (STAT) proteins, peroxisome proliferator‐activated receptor gamma (PPAR‐γ), and interferon regulatory factors (IRFs). Various signaling pathways, such as the Toll‐like receptor (TLR) pathway, the NLRP3 inflammasome pathway, and the JAK–STAT pathway, are involved in the regulation of microglial and macrophage properties. Besides, epigenetic regulation of the transcription factors and molecular pathways, such as DNA methylation and histone acetylation, can influence the activity of transcription factors involved in the phenotypic shift of microglia and macrophages. For example, epigenetic regulation of NF‐κB and STAT signaling pathways can impact the expression of proinflammatory genes of microglia and macrophages. However, the particular mediator that tips the balance from anti‐inflammatory phenotype toward proinflammatory subsets at around 7 days after ischemic stroke in microglia and the infiltrated macrophages remains elusive. Here we found that STING activation is a key driver of the phenotypic shift. Overloading of lysosomes in microglia and macrophages facilitates the escape of STING from degradation, leading to the activation of the type I interferon signaling pathway and the augmentation of their proinflammatory properties. In the management of the functional properties of microglia and macrophages in stroke lesion, transcriptional signaling pathways interact with each other and with various environmental cues. How STING and type I interferon signaling pathway orchestrate the activation of other transcriptional factors and molecular pathways to govern the activities of microglia and macrophages remains to be studied.

Aging is one of the most critical risk factors of ischemic stroke. Research has indicated that the activation of STING and type I interferon signaling may undergo changes during aging. While the exact nature of these alterations is still an active area of investigation, there is evidence to suggest that the activation of these pathways may be dysregulated in older individuals. Dysregulation of STING activation when confronting cytosolic DNA in aged cells has been linked to impaired antiviral responses and increased susceptibility to certain infections. Similarly, dysregulated activation of type I interferon signaling in aging has been associated with impaired immune function and increased susceptibility to viral infections. Conversely, “inflammaging,” which refers to the chronic, low‐grade inflammation associated with aging, has been partially attributed to prolonged activation of STING and type I interferon signaling in aged individuals. The alteration of STING and type I interferon signaling in senescent microglia and macrophages after stroke and subsequent impacts on stroke outcomes are questions to be answered.

By investigating the impact of inhibiting STING activation on the phenotypic shift of microglia and macrophages toward a proinflammatory phenotype after prolonged phagocytosis, we explored the potential therapeutic interventions. The therapeutic efficacy of H‐151 in stroke models was assessed, revealing improved disease outcomes even with delayed treatment. These findings suggest that to suppress STING signaling inhibits the phenotypic shift of microglia and macrophages toward a proinflammatory phenotype, subsequently ameliorating neural inflammation and providing protection against ischemic stroke. We thus propose that inhibiting STING and subsequent activation of type I interferon signaling is a promising therapeutic strategy in sub‐acute phase of ischemic stroke.

Conclusively, the study provides novel insights into the interplay between lysosomal function, STING activation, and the type I interferon signaling pathway in the context of acute ischemic stroke. Our findings have the potential to inform the development of targeted strategies for mitigating neuroinflammatory responses and ameliorating poststroke neural inflammation, with broader implications for the treatment of neuroinflammatory conditions and extended therapeutic time window.

## 4 Methods

### 4.1 Study Approval

The animal experimental studies were approved by the Medical Ethics Committee of the Third Affiliated Hospital of Sun Yat‐sen University and the Animal Care and Use Committee of Sun Yat‐Sen University.

### 4.2 Animals

C57BL/6 wild‐type (WT) mice (8 weeks old, weight 18–25 g) were purchased from the Guangdong Medical Laboratory Animal Center (Guangzhou, China) and housed in a humidity‐ and temperature‐controlled animal facility at the Sun Yat‐sen University with a 12‐h light–dark cycle. The mice were given access to normal chow and tap water ad libitum.

### 4.3 Quality Control Analysis of scRNA‐Seq Datasets

Seurat, a popular R package, was used for quality control (QC) using the following criteria: (1) gene counts between 200 and 7000; (2) cells with a mitochondrial gene expression ratio ≤ 40%. The filtered gene barcode matrices were then processed for dimensionality reduction, clustering, and visualization.

### 4.4 Single‐Cell RNA‐Seq Data Analysis

We used Seurat (version 4.3.1) for downstream analysis, following these seven steps: (1) Counts were log normalized for each cell using the natural logarithm of 1 + counts per 10,000. (2) The 2000 most variable genes were identified using the FindVariableFeatures function. (3) Expression values for each gene across all cells were standardized through *Z*‐score transformation (ScaleData). (4) Principal component analysis (PCA) was performed on the scaled variable gene matrix, and batch correction was applied using FindIntegrationAnchors and IntegrateData, incorporating Sham, tMCAO5d, and tMCAO14d samples. (5) UMAP was utilized for dimensional reduction and visualization of PCA‐derived embeddings in two‐dimensional space, using the first 15 PCA components. (6) The Louvain algorithm, implemented in FindClusters with a resolution of 0.6, was used for graph‐based clustering on the neighbor graph constructed from PCA‐derived embeddings via the FindNeighbors function. (7) After clustering, the model‐based analysis of single‐cell transcriptomics (MAST) algorithm (version 1.26) was employed in the FindAllMarkers function to identify differentially expressed genes (DEGs) in each cluster, based on the log‐normalized expression matrix with parameters: pos = TRUE, min.pct = 0.5, logfc.threshold = log2(1.5), and max.cells.per.ident = 2000.

### 4.5 Differential Expression Analysis and Cell Type Identification

We used the SingleR package (version 2.2.0) and referenced established marker genes from the literature [16] to define clusters into 10 major cell types: macrophages, microglia, DC cells, lymphocytes, neutrophils, oligodendrocytes, astrocytes, neurons, vascular cells, and ependymal cells. Simultaneously, scRNAtoolVis (0.0.7) was used to create heatmap visualizations of the marker genes for each cell type.

### 4.6 Gene Enrichment and Pathway Analysis

In this study, the FindMarkers function with parameters min.pct = 0.01 and logfc.threshold = 0 was used to obtain a ranked list of all genes based on their expression levels. Comparisons included: (1) proinflammatory phenotype versus inflammatory resolving phenotype of macrophages, (2) proinflammatory phenotype versus inflammatory resolving phenotype of microglia, (3) macrophages at tMCAO 14 days versus macrophages at tMCAO 5 days, and (4) microglia at tMCAO 14 days versus microglia at tMCAO 5 days. Gene sets from the Gene Ontology (GO) database were used for GSEA using clusterProfiler, with final visualization using GseaVis. The focus was primarily on GO Biological Processes.

### 4.7 Trajectory Analysis

Genes from macrophages/microglia were input into the Monocle2 R package (version 2.14.0, https://bioconductor.org/packages/release/bioc/html/monocle.html) for trajectory analysis. Dimensionality reduction was conducted using the DDRTree method with default parameters. Branch expression analysis modeling (BEAM) in Monocle 2 identified specific genes enriched along particular branches in the pseudotime tree. Branched heatmaps were created with the genes of interest. The orderCells function was analyzed using the root state argument to specify the homeostatic macrophages/microglia branch as the starting point of the trajectory.

### 4.8 Model of Acute Ischemic Stroke and Drug Administration

Mice were subjected to focal acute ischemic stroke induced by transient middle cerebral artery occlusion (tMCAO). Procedures for tMCAO were described previously [17]. Briefly, mice were anesthetized with 1.5%–2.0% isoflurane under spontaneous breathing conditions. A filament was inserted into the external carotid artery (ECA) and directed to the middle cerebral artery (MCA) through the internal carotid artery (ICA). Filament insertion into the ICA was maintained for 60 min followed by reperfusion, maintaining core body temperatures. Cerebral blood flow (CBF) during surgery was measured by laser Doppler flow cytometry. Mice with a more than 70% reduction in blood flow in the ischemic core were included in the study; mice that died during surgery were excluded. Sham‐operated animals underwent a similar procedure without filament insertion. All efforts were made to minimize animal suffering. tMCAO mice were randomly assigned to receive vehicle or H‐151 treatment once daily for 10 days, beginning 5 days after reperfusion. Control animals received an equal volume of vehicle control (20% sulfobutylether‐β‐cyclodextrin) using the same anesthesia and injection method. In this study, H‐151 (10 mg/kg) was administered i.p. as a suspension in 20% sulfobutylether‐β‐cyclodextrin [13]. Mouse survival was recorded.

### 4.9 Measurement of Infarct Volume

Six equally spaced coronal brain sections encompassing the MCA territory were stained with an antimicrotubule‐associated protein 2 (NeuN, a neuron‐specific marker) antibody (ab177487, 1:1000; Abcam) to visualize live brain tissue. Infarct volume was analyzed by a blinded observer using NIH ImageJ software on NeuN‐stained sections. Infarct volume with corrections for edema was calculated as the volume of the contralateral hemisphere minus the noninfarcted volume of the ipsilateral hemisphere.

### 4.10 Primary Mouse BMDM Culture

Primary BMDMs cultures were prepared by isolating cells from the femurs and tibiae of healthy WT C57/BL6 WT donors (8–12 weeks old). The macrophage precursors were cultivated and differentiated into macrophages over 6 days in MCSF‐conditioned RPMI1640 medium supplemented with 10% FBS and 1% penicillin–streptomycin (PS).

### 4.11 Primary Mouse Microglia Culture

Primary microglia were prepared from the whole brains of postnatal days 1–3 mouse pups. Briefly, brain tissue was isolated and digested using trypsin, then resuspended in DMEM medium (Gibco 11965118) supplemented with 10% FBS (Gibco 1921005PJ). The cell suspensions were incubated in tissue flasks pretreated with poly‐d‐lysine (Gibco A3890401). After 4–7 days, astrocytes proliferated, and microglia were generated by adding DMEM medium containing 20% L929‐conditioned medium. Three to four days later, microglia were isolated from mixed glial cell cultures by shaking at 180 rpm for 1 h. Finally, the microglia were resuspended in RPMI containing 20% L929 conditioned medium.

### 4.12 Preparation of Danger‐Associated Molecular Patterns

Danger‐associated molecular patterns (DAMPs) were extracted from brain tissue of C57/BL6 WT donors as previously described [18]. Briefly, a brain hemisphere was chopped and digested with 1 mL of 0.25% trypsin–EDTA (Thermo Fisher Scientific, 25300054) for 20 min at 37°C. Digestion was terminated by adding 2 mL of culture medium (RPMI1640 + 10% FBS + 1% PS). The brain lysate was then subjected to centrifugation (10,000 *g*, 30 min) to remove brain cells. The supernatant was collected and filtered through a 0.22‐μm filter. Two parts of DAMPs were applied to the culture system with eight parts of BMDM culture medium for the indicated time period.

### 4.13 Behavioral Tests

Sensorimotor functions were assessed with the rotarod, foot fault, and adhesive removal tests conducted 1–3 days prior to and 1–14 days following tMCAO. Cognitive function was evaluated using the Morris water maze test 10–14 days after tMCAO. Affective disorders were examined through the open field test and novel object recognition test conducted 14 days post‐tMCAO.

### 4.14 Rotarod Test

Mice underwent a three‐day pretraining period using an accelerated rotarod paradigm, with data from the final session serving as baseline measurements. Performance tests were conducted at 5, 7, 10, and 14 days post‐tMCAO. During each trial, mice were placed on a rotating drum that accelerated from 0 to 300 rpm over 6 min, maintaining this final speed. A trial concluded if a mouse either fell off the drum or spun around for two consecutive revolutions without attempting to walk. The latency to fall or spin was recorded, and data were expressed as mean values from three trials.

### 4.15 Foot Fault Test

Each mouse was placed on a stainless steel grid floor (20 cm × 40 cm with a mesh size of 4 cm^2^) elevated 1 m above the floor. The total number of steps was counted during a videotaped 1‐min observation period. The number of forelimb foot faults (when the forelimb fell through the grid) was recorded. Mice were pretrained for 3 consecutive days before tMCAO, with data from the last training session recorded as baseline. Tests were performed at 5 days, 7 days, 10 days, and 14 days after tMCAO.

### 4.16 Adhesive Removal Test

The adhesive removal test was performed to assess tactile responses and sensorimotor asymmetries. A 2 × 3 mm adhesive tape was applied to the right forepaws. Tactile responses were measured by recording the time to touch and remove the adhesive tape. The maximum observation period was 120 s. Each training/testing day included three trials per mouse. The mean values were recorded as data. Mice were pretrained for 3 consecutive days before Bevacizumab administration, with data from the last training session set as baseline. Tests were performed at poststroke 5 days, 7 days, 10 days, and 14 days.

### 4.17 Morris Water Maze Test

Cognitive function was assessed using the Morris water maze test 10–14 days after tMCAO. In the learning test, a square platform (11 × 11 cm^2^) was submerged 2 cm below the water surface in a circular pool (150 cm in diameter) filled with opaque water. Mice were placed into the pool from one of four locations and given 60 s to locate the hidden platform. Each mouse underwent four trials per day with randomly assigned starting positions for 4 consecutive days, beginning 10 days post‐tMCAO. At the end of each trial, the mouse was placed on the platform or allowed to stay there for 30 s, with prominent spatial cues visible in the room. The time taken to reach the platform was recorded during the learning phase. On day 14, the platform was removed, and a single 60‐s probe trial was conducted to record the time spent in the goal quadrant, where the platform had previously been located.

### 4.18 Open Field Test

The open‐field arena was a white box measuring 40 × 40 × 40 cm, illuminated with red light (80–100 Lux) and positioned inside a sound‐insulated chamber. Mice were placed in a corner of the arena and allowed to explore for 5 min. The total distance traveled, along with the distance covered and time spent in the 20 × 20 cm center zone, were monitored and analyzed.

### 4.19 Novel Object Recognition Test

The novel object recognition test was conducted to evaluate the object recognition memory of mice. One hour before the test, each mouse was allowed to explore two identical objects. One of these objects was then replaced with a novel object that differed in shape and color but was roughly the same height and volume. Mice typically exhibit an innate preference for novelty, spending more time exploring novel objects compared to familiar ones. The time spent exploring the familiar and novel objects was recorded using EthoVision XT. Both the recognition index (RI) and discrimination index (DI) were used to assess mouse performance. RI was calculated as the time spent exploring the new object divided by the total exploration time, while DI was calculated using the formula: (new object exploration time − familiar object exploration time)/total exploration time.

### 4.20 Immunofluorescence Staining

Animals were euthanized and perfused with PBS followed by 4% paraformaldehyde. After sufficient perfusion, brains were removed and cut into 25‐μm frozen cryosections using a microtome. Brain sections were incubated with primary antibodies at 4°C overnight. After washing with PBS, sections were incubated with secondary antibodies for 1 h at room temperature. Sections were then washed and mounted with mounting medium containing DAPI (Abcam, ab104139). The following primary antibodies were used: rabbit anti‐Iba1 (1:1000, 019‐19741, Wako Pure Chemical Industries), rabbit anti‐IFN‐γ (1:500, 15365‐1‐AP, Proteintech), rabbit anti‐IL10 (1:500, 20850‐1‐AP, Proteintech), rabbit anti‐TNF‐α (1:500, 17590‐1‐AP, Proteintech), rabbit anti‐CD206 (1:500, ab125028, Abcam), rabbit anti‐STING (1:500, 19851‐1‐AP, Proteintech), rabbit anti‐pIRF3 (1:400, AF2436, Affinity), rabbit anti‐NeuN (1:1000, ab177487, Abcam), and rabbit anti‐dMBP (1:2000, AB5864, Affinity). The following secondary antibodies were applied: anti‐rabbit secondary antibody conjugated with Cy3 (1:1000, 111‐165‐003, Jackson ImmunoResearch Laboratories), anti‐rabbit secondary antibody conjugated with Alexa Fluor 488 (1:1000, 711‐545‐152, Jackson ImmunoResearch Laboratories). Phalloidin (Invitrogen A12379, 1:3000) was used to label actin to outline cells when indicated. Confocal microscopy images were acquired using a Leica SP confocal microscope and Leica confocal software. Immunopositive cell quantification and area analysis were performed using NIH ImageJ software by an investigator blinded to the experimental design.

### 4.21 Multiplex Immunohistochemistry (MIHC)

MIHC was used to detect multiple markers on a single section simultaneously. Brain sections were incubated with primary antibodies at 4°C overnight. After washing with PBS, the sections were incubated with a mouse‐rabbit secondary antibody (1:10, 10,013,001,010, PANOVUE) at room temperature for 10 min. After another PBS wash, the sections were incubated in a solution of monochromatic TSA fluorescent dye (10,008,100,100, PANOVUE), first diluted 1:100 in TSA amplification reagent (10,021,001,010, PANOVUE), then vortexed and further diluted 1:10 in PBS for 10 min. The sections were subsequently treated with antigen retrieval solution (10,108,001,010, PANOVUE) at room temperature for 10 min, followed by staining for the next marker using the aforementioned immunofluorescence procedure.

### 4.22 Flow Cytometric Analysis

In vitro, microglia or macrophages were digested with 0.25% trypsin–EDTA (Thermo Fisher Scientific, Carlsbad, CA, USA) to prepare single‐cell suspensions for flow cytometry. After washing with PBS, the cells were fixed and permeabilized using the Invitrogen Intracellular Fixation & Permeabilization Buffer Set for subsequent staining. For in vivo experiments, brain tissue was homogenized into single‐cell suspensions for flow cytometry (FACS). The brains were dissected and the ipsilateral hemispheres were collected. Each hemisphere was digested with 0.25% trypsin–EDTA at 37°C for 25 min, then passed through a 70‐μm cell strainer. Brain cells were separated from myelin debris by centrifugation using a 30%/70% Percoll solution (GE Healthcare Biosciences AB, Uppsala, Sweden). The interface cells were collected, washed with PBS, fixed, permeabilized, and stained. The following antibodies were used: CD45‐Percp (1:400, BioLegend), CD11b‐PECY7 (1:400, BioLegend), F4/80‐488 (1:400, BioLegend), F4/80‐APC (1:400, BioLegend), IL10‐PE (1:200, BioLegend), TNF‐α‐FITC (1:200, BioLegend), and IFN‐γ‐APC (1:200, BioLegend). Flow cytometric analysis was performed using a flow cytometer (Agilent, NovoCyte Advanteon), and data were analyzed using FlowJo X 10.8.1 software. Appropriate isotype controls were stained following the manufacturer's instructions (Thermo Fisher Scientific, Carlsbad, CA, USA). Fluorochrome compensation was performed using single‐stained OneComp eBeads (Thermo Fisher Scientific). For data presentation, when cells could be divided into negative or positive populations, the percentage of cells was calculated. When marker expression was continuous and population separation was unclear, data were presented as mean fluorescence intensity (MFI).

### 4.23 Western Blot

Protein isolation was performed as previously described [19]. Western blots were performed using standard SDS‐polyacrylamide gel electrophoresis and enhanced chemiluminescence detection reagents (GE Healthcare Biosciences AB, Uppsala, Sweden). Antibodies against STING (1:1000, 19851‐1‐AP, Proteintech), pIRF3 (1:500, AF2436, Affinity), pIRF7 (1:500, AF8486, Affinity), pTBK1 (1:500, AF8190, Affinity), TBK1 (1:500, DF7026, Affinity), IRF3 (1:500, DF6895, Affinity), IRF7 (1:500, DF7503, Affinity), IFNB1 (1:400, DF6471, Affinity), and GAPDH (1:2000, 21,185, Cell Signaling Technology) were used according to the manufacturer's directions. Immunoreactivity was assessed using ImageJ (NIH).

### 4.24 Quantitative Determination of mRNA Expression

Total RNA was extracted from cells using a commercial kit (ESscience) following the manufacturer's instructions. For first‐strand cDNA synthesis, 1 μg of RNA (OD 260 nm/280 nm = 1.8–2.2) was used in a 40‐μL reaction with a PrimeScript RT reagent kit (Takara). Real‐time polymerase chain reaction (RT‐PCR) was conducted on a QuantStudio 5 (ABI) using a TB Green Premix Ex Taq kit (Takara), with 1 μL of synthesized cDNA and ROX added to each reaction. The cycling conditions included: 95°C for 30 s; 95°C for 5 s and 60°C for 34 s, repeated for 40 cycles; followed by a melt curve analysis at 95°C for 15 s, 60°C for 1 min, and 95°C for 15 s. Primers used in the study are listed in Table S1. The double delta CT method was employed, and data are presented as fold changes normalized to PBS‐treated macrophages. Glyceraldehyde‐3‐phosphate dehydrogenase (GAPDH) served as the housekeeping gene for normalization.

### 4.25 Phagocytosis Assay

DAMPs extracted from naïve mouse brains were administered to primary macrophages and microglia for 48 h. H‐151 (1 μmol/L) was applied to these cells for the same duration. To assess phagocytic activity, the cells were then cocultured with either fluorescein‐labeled *Escherichia coli* (EZCell Phagocytosis Assay Kit, Red *E. coli*, BioVision) or fluorescein‐labeled β‐amyloid protein (20 μg/mL, β‐Amyloid [1–40], FAM‐labeled TFA, MCE) for 1 h. The control group consisted of either a blank control or a vehicle control. The fluorescence intensity of *E. coli* or β amyloid is detected by flow cytometry or immunofluorescence.

### 4.26 Statistical Analysis

Normality tests were conducted prior to performing parametric analyses using Student's *t* test and one‐way ANOVA, followed by Tukey's post hoc test. Statistical analyses were carried out using GraphPad Prism software (version 8.0), with significance set at *p* < 0.05. An unpaired, two‐tailed Student's *t* test was used to compare data between two groups. Error bars represent the standard error of the mean (SEM). For comparisons among three or more groups, one‐way ANOVA (without matching or pairing) was applied, and results were adjusted for multiple comparisons using Dunnett's test. Error bars also represent SEM.

## Author Contributions

**Zhiruo Liu** designed and performed the experiments, collected and analyzed the data, and drafted the manuscript. **Qin Qin** contributed to the experimental design and revised the manuscript. **Shisi Wang** and **Xinmei Kang** performed the animal experiments and collected the data. **Yuxin Liu** and **Lei Wei** contributed to the experimental design and manuscript preparation. **Zhengqi Lu**, **Mengyan Hu**, and **Wei Cai** designed and supervised the study and critically revised the manuscript. All authors read and approved the final manuscript.

## Ethics Statement

All animal experiments were approved by the Third Affiliated Hospital of Sun Yat‐sen University and conducted in accordance with the Guide for the Care and Use of Laboratory Animals and Stroke Treatment guidelines.

## Consent

The authors have nothing to report.

## Conflicts of Interest

The authors declare no conflicts of interest.

## Supporting information


**Figure S1.** Identification of brain cell clusters and activated biological processes in macrophages poststroke. Brain cells were clustered and annotated. Biological processes in microphages were analyzed by gene set enrichment analysis (GSEA) poststroke. (A) Identification of 10 cell clusters based on established marker genes. GSEA comparing macrophages 14 days and 5 days poststroke. (B) Activated biological processes in macrophages were presented. Pathway related to the response to interferon β was highlighted. (C) Running enrichment scores for the listed biological processes.
**Figure S2.** Phenotype shift in microglia during stroke progression. Microglia were categorized and differentially expressed genes in proinflammatory microglia were analyzed by gene set enrichment analysis (GSEA). (A) Uniform Manifold Approximation and Projection (UMAP) division of microglia into 18 distinct subclusters. (B) Top three marker genes for each microglia cluster and average expression of classic inflammatory related genes. (C) Identification of three main microglia subtypes during stroke progression. GSEA of differentially expressed genes in proinflammatory microglia. (D) Activated biological processes were presented. (E) Running enrichment scores for the listed biological processes. GSEA of differentially expressed genes in microglia 14 days and 5 days poststroke. (F) Activated biological processes was presented. Pathway related to the response to interferon β was highlighted. (G) Running enrichment scores for the listed biological processes. (H) Pseudotime trajectory of microglia throughout the stroke timeline. (I) Along the pseudotime trajectory, microglia display a variable expression pattern of both classical anti‐inflammatory and proinflammatory genes, alongside genes implicated in the STING (stimulator of interferon genes) pathway.
**Figure S3.** Activation of the STING (stimulator of interferon genes) signaling pathway in macrophages/microglia after stroke. (A) Male C57/BL6J mice (aged 8–12 weeks) were subjected to 60 min of transient middle cerebral artery occlusion (tMCAO), and brain tissue was collected for immunostaining at specified time points. Protein expression of STING and nuclear translocation of phospho‐interferon regulatory factor 3 (pIRF3) were analyzed. *N* = 6 per group, **p < 0.01, ***p < 0.001; by one‐way ANOVA. A mixture of brain‐derived danger‐associated molecular patterns (DAMPs) was extracted from naïve mouse brains and administered to primary macrophages and microglia for short‐term (24 h) and prolonged (48 h) phagocytosis. Following treatment, we assessed the inflammatory properties and STING activation in microglia and macrophages. (B) Western blot analysis revealed activation of the STING and type I interferon pathways in macrophages subjected to prolonged DAMPs phagocytosis. Experiments were conducted three times. *p < 0.05; by one‐way ANOVA. (C) Immunostaining experiments showed protein expression of STING and the nuclear translocation of pIRF3, with statistical significance as noted. Experiments were conducted four times. **p < 0.01, ***p < 0.001; by one‐way ANOVA.
**Figure S4.** STING (stimulator of interferon genes) inhibitor H‐151 restores the phagocytic function in macrophages/microglia. A mixture of brain‐derived danger‐associated molecular patterns (DAMPs) was extracted from naïve mouse brains and administered to primary macrophages and microglia for prolonged (48 h) phagocytosis. The specific inhibitor of STING, H‐151(1 μmol/L), was applied to microglia and macrophages for 48 h. Primary macrophages or microglia were cocultured with either fluorescein‐labeled *Escherichia coli* or β‐amyloid protein for 1 h. Immunostaining analysis and flow cytometry were conducted on macrophages (A) and microglia (B). Experiments were conducted four times. *p < 0.05, ***p < 0.001; by one‐way ANOVA.
**Figure S5.** STING (stimulator of interferon genes) inhibitor H‐151 improves stroke outcome. Male C57/BL6J mice (aged 8–12 weeks) were subjected to 60 min of tMCAO. H‐151 (10 mg/kg) was administered to mice from days 5 to 14 after tMCAO via intraperitoneal injection (i.p.), once daily (qd). The severity of the stroke condition was assessed. (A) A schematic diagram of the experimental strategy. (B) Statistical analysis of the blood flow data in Figure 6A. *N* = 10 in each group. (C) Statistical analysis of the infarct volume in Figure 6B. *N* = 10 in each group. ***p < 0.001; by one‐way ANOVA. (D) Mice were subjected to novel object recognition tests (trained and tested 10 days after tMCAO). Motor activity and the time spent exploring familiar (*F*) and novel (*N*) objects were quantified. Discrimination score (*N*−*F*) and discrimination ratio (*N*/[*N* + *F*]) were calculated. *N* = 10 in each group. ***p < 0.001; by one‐way ANOVA.
**Table S1.** Primers used in the study.

## Data Availability

All data needed to evaluate the conclusions of this article are provided within the main text and the Supporting Information. The datasets used and analyzed in this study are available from the corresponding author upon reasonable request. Public databases GSE227651 and GSE171171 were also used for the data analysis in this study.
