# Supplementary material for: STING Activation in Macrophages and Microglia Drives Poststroke Inflammation: Implications for Neuroinflammatory Mechanisms and Therapeutic Interventions
Source: CNS Neurosci Ther. 2024 Dec 19;30(12):e70106. doi: 10.1111/cns.70106 (PMC11656094; doi:10.1111/cns.70106)
Supplement: Supplementary file 1 — Figure S1. Identification of brain cell clusters and activated biological processes in macrophages poststroke. Brain cells were clustered and annotated. Biological processes in microphages were analyzed by gene set enrichment analysis (GSEA) poststroke. (A) Identification of 10 cell clusters based on established marker genes. GSEA comparing macrophages 14 days and 5 days poststroke. (B) Activated biological processes in macrophages were presented. Pathway related to the response to interferon β was highlighted. (C) Running enrichment scores for the listed biological processes. Figure S2. Phenotype shift in microglia during stroke progression. Microglia were categorized and differentially expressed genes in proinflammatory microglia were analyzed by gene set enrichment analysis (GSEA). (A) Uniform Manifold Approximation and Projection (UMAP) division of microglia into 18 distinct subclusters. (B) Top three marker genes for each microglia cluster and average expression of classic inflammatory related genes. (C) Identification of three main microglia subtypes during stroke progression. GSEA of differentially expressed genes in proinflammatory microglia. (D) Activated biological processes were presented. (E) Running enrichment scores for the listed biological processes. GSEA of differentially expressed genes in microglia 14 days and 5 days poststroke. (F) Activated biological processes was presented. Pathway related to the response to interferon β was highlighted. (G) Running enrichment scores for the listed biological processes. (H) Pseudotime trajectory of microglia throughout the stroke timeline. (I) Along the pseudotime trajectory, microglia display a variable expression pattern of both classical anti‐inflammatory and proinflammatory genes, alongside genes implicated in the STING (stimulator of interferon genes) pathway. Figure S3. Activation of the STING (stimulator of interferon genes) signaling pathway in macrophages/microglia after stroke. (A) Male C57/BL6J mice [file CNS-30-e70106-s001.docx]

**Supplemental Material**

**Supplemental Figures**

**
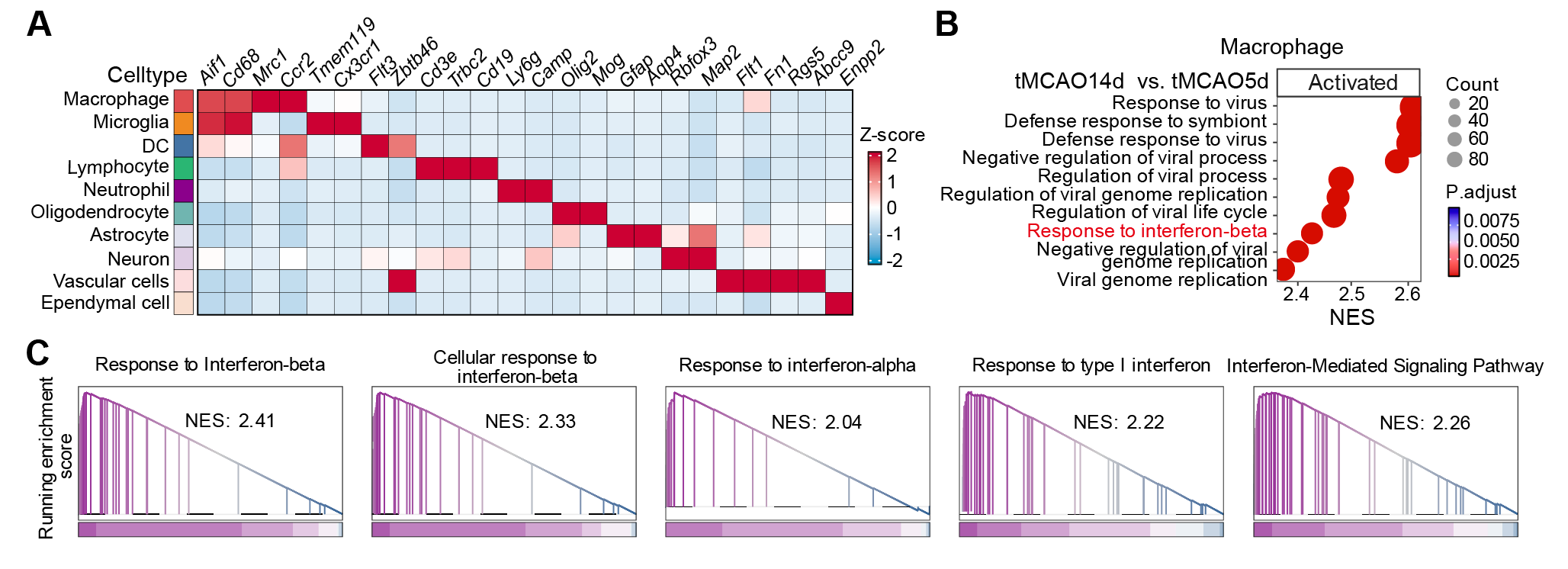
 Figure S1. Identification of brain cell clusters and activated biological processes in macrophages post-stroke**

Brain cells were clustered and annotated. Biological processes in microphages were analyzed by GESA post-stroke. (**A**) Identification of 10 cell clusters based on established marker genes. (**B-C**) GSEA analysis comparing macrophages 14d and 5d post-stroke. (**B**) Activated biological processes in macrophages were presented. Pathway related to the response to interferon-beta was highlighted. (**C**) Running enrichment scores for the listed biological processes.


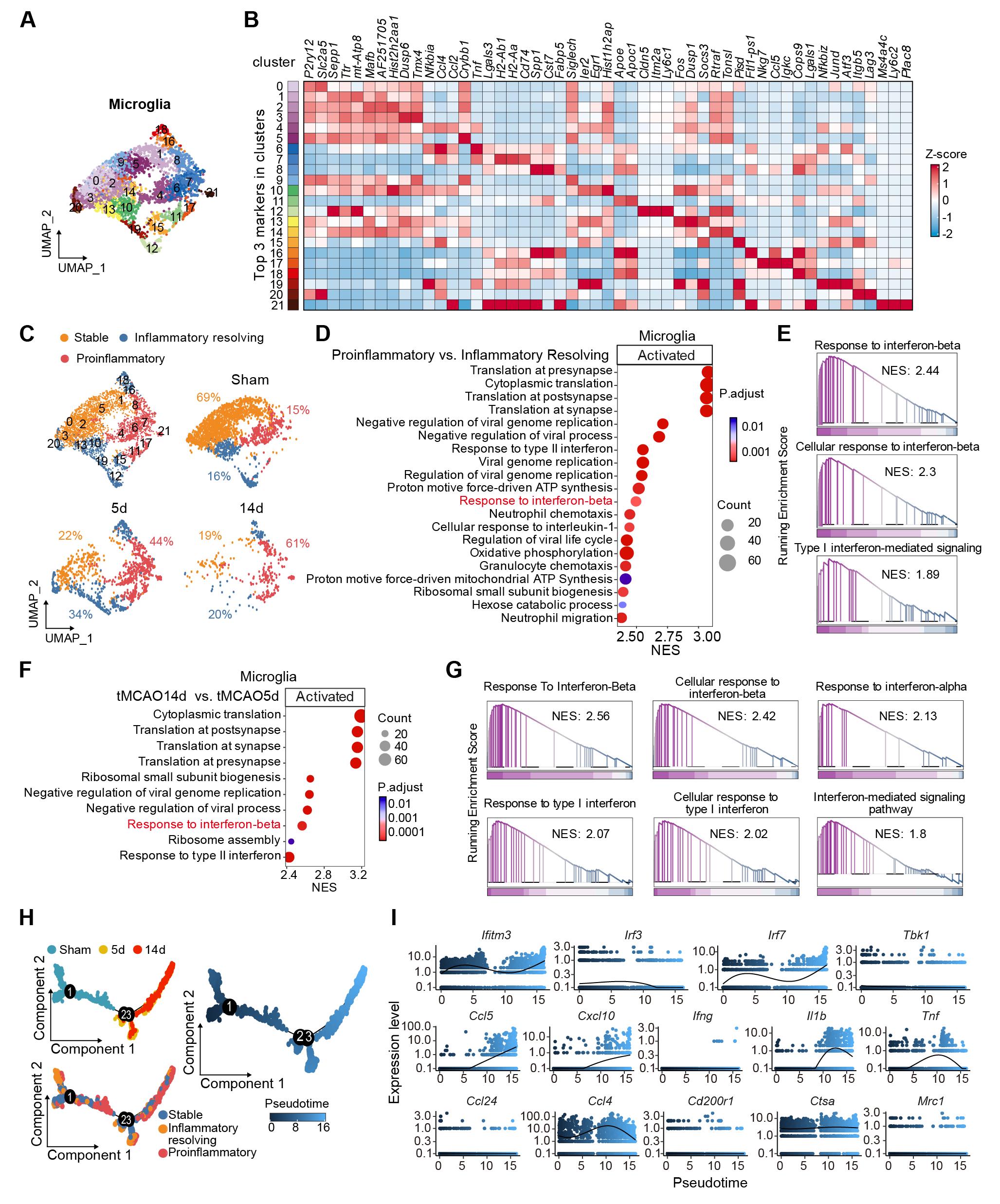


**Figure S2.** **Phenotype shift in microglia during stroke progression**

Microglia were categorized, and differentially expressed genes in pro-inflammatory microglia were analyzed by GSEA. (**A**) UMAP division of microglia into 18 distinct subclusters. (**B**) Top 3 marker genes for each microglia cluster and average expression of classic inflammatory-related genes. (**C**) Identification of three main microglia subtypes during stroke progression. (**D-E**) GSEA of differentially expressed genes in pro-inflammatory microglia. (**D**) Activated biological processes were presented. (**E**) Running enrichment scores for the listed biological processes. (**F-G**) GSEA of differentially expressed genes in microglia 14d and 5d post-stroke. (**F**) Activated biological processes was presented. Pathway related to the response to interferon-beta was highlighted. (**G**) Running enrichment scores for the listed biological processes. (H) Pseudotime trajectory of microglia throughout the stroke timeline. (**I**) Along the pseudotime trajectory, microglia display a variable expression pattern of both classical anti-inflammatory and pro-inflammatory genes, alongside genes implicated in the STING pathway.

**
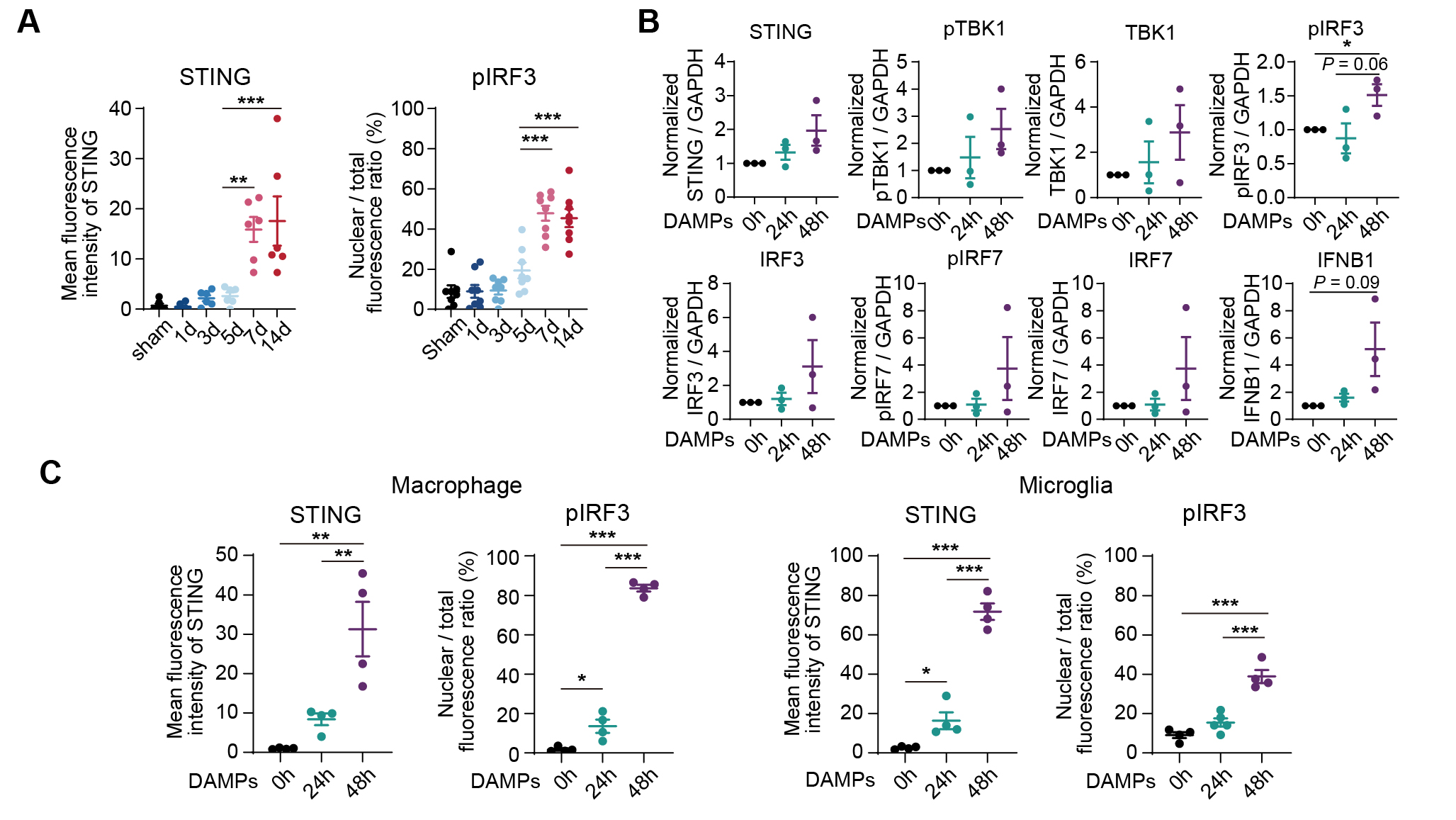
**

**Figure S3. Activation of the STING signaling pathway in macrophages/microglia after stroke.**

(**A**) Male C57/BL6J mice (age 8-12 weeks) were subjected to 60 minutes of tMCAO, and brain tissue was collected for immunostaining at specified time points. Protein expression of STING and nuclear translocation of pIRF3 were analyzed. *N* = 6 per group, ***P* < 0.01, ****P* < 0.001; by one-way ANOVA. (**B-C**) A mixture of brain-derived danger-associated molecular patterns (DAMPs) was extracted from naïve mouse brains and administered to primary macrophages and microglia for short-term (24 hours) and prolonged (48 hours) phagocytosis. Following treatment, we assessed the inflammatory properties and STING activation in microglia and macrophages. (**B**) Western blot analysis revealed activation of the STING and type I interferon pathways in macrophages subjected to prolonged DAMPs phagocytosis. Experiments were conducted three times. **P* < 0.05; by one-way *ANOVA*. (**C**) Immunostaining experiments showed protein expression of STING and the nuclear translocation of pIRF3, with statistical significance as noted. Experiments were conducted four times. ***P* < 0.01, ****P* < 0.001; by one-way *ANOVA*.

**
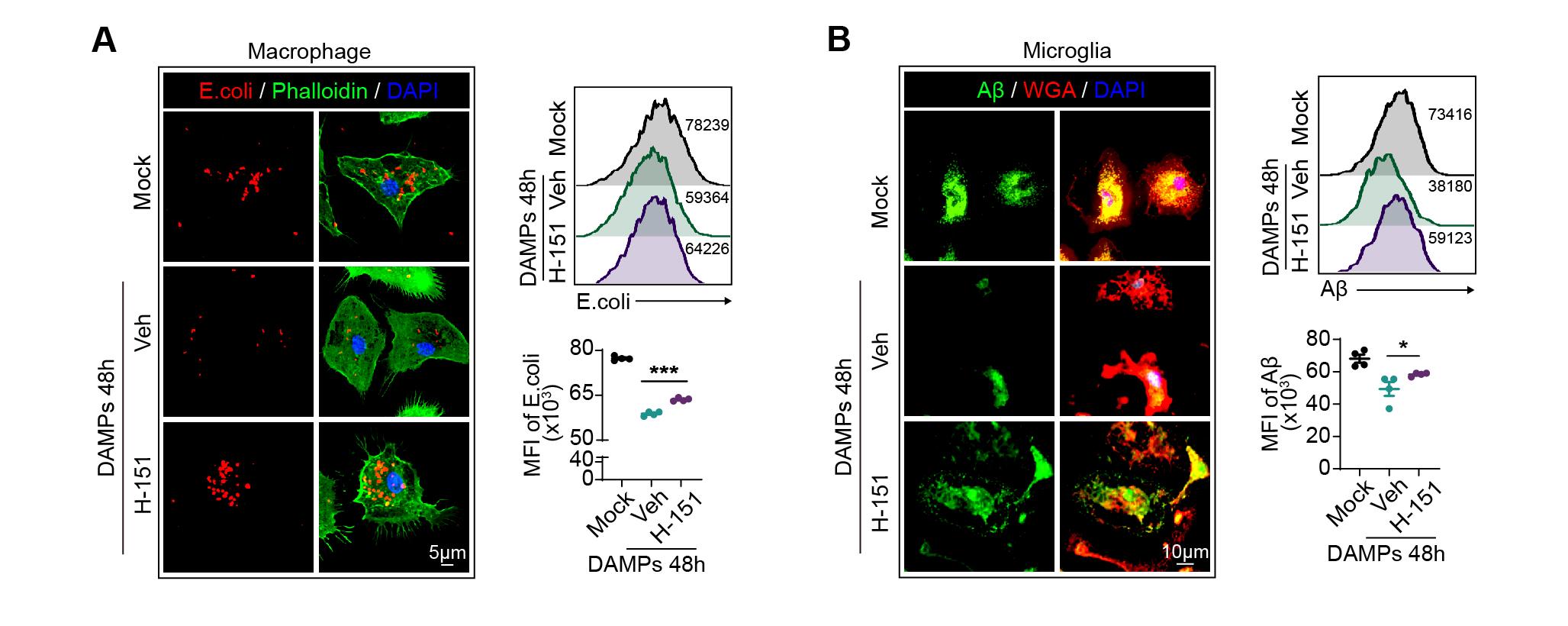
**

**Figure S4. STING inhibitor H-151 restores the phagocytic function in macrophages/microglia**

A mixture of brain-derived danger-associated molecular patterns (DAMPs) was extracted from naïve mouse brains and administered to primary macrophages and microglia for prolonged (48 hours) phagocytosis. The specific inhibitor of STING, H-151(1 umol/L), was applied to microglia and macrophages for 48 hours. Primary macrophages or microglia were co-cultured with either fluorescein-labeled E. coli or β-amyloid protein for one hour. Immunostaining analysis and flow cytometry were conducted on macrophages (**A**) and microglia (**B**). Experiments were conducted four times. **P* < 0.05, ****P* < 0.001; by one-way *ANOVA*.

**
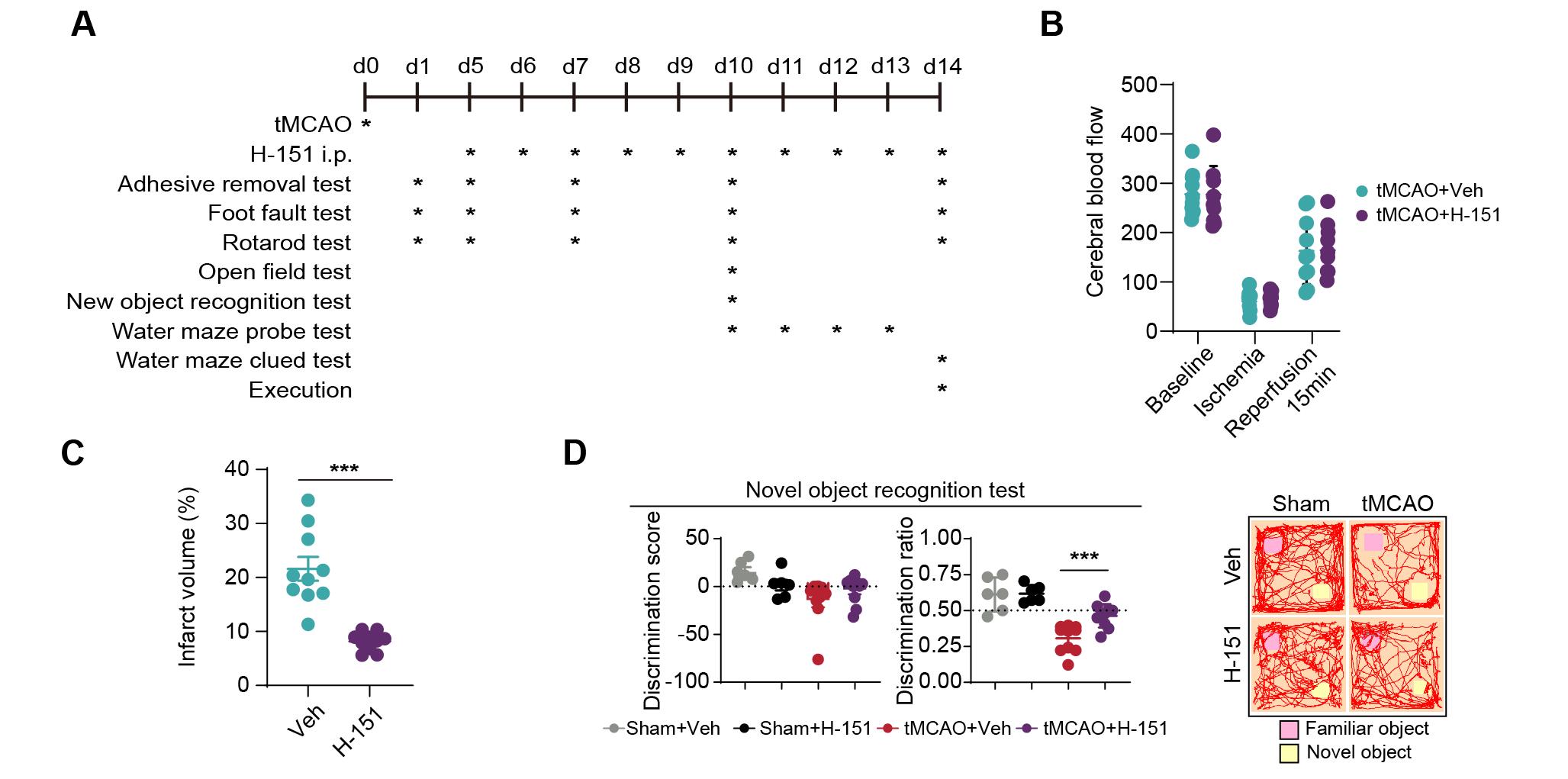
**

**Figure S5. STING inhibitor H-151 improves stroke outcome**

Male C57/BL6J mice (age 8-12 weeks) were subjected to 60 minutes of tMCAO. H-151 (10 mg/kg) was administered to mice from day 5 to 14 days after tMCAO via intraperitoneal injection (i.p.), once daily (qd). The severity of the stroke condition was assessed. (**A**) A schematic diagram of the experimental strategy. (**B**) Statistical analysis of the blood flow data in **Fig. 6A**. *N* = 10 in each group. (**C**) Statistical analysis of the infarct volume in **Fig. 6B**. *N* = 10 in each group. ****P* < 0.001; by one-way *ANOVA*. (**D**) Mice were subjected to novel object recognition tests (trained, and tested 10 days after tMCAO). Motor activity and the time spent exploring familiar (F) and novel (N) objects were quantified. Discrimination score (N-F) and discrimination ratio ((N / (N+F)) were calculated. *N* = 10 in each group. ****P* < 0.001; by one-way *ANOVA*.  **Supplemental Table**

Table 1. Primers used in the study

| Gene | Forward primer | Reverse primer |
| --- | --- | --- |
| *Il4* | GGTCTCAACCCCCAGCTAGT | GCCGATGATCTCTCTCAAGTGAT |
| *Il1a* | AAGACAAGCCTGTGTTGCTGAAGG | TCCCAGAAGAAAATGAGGTCGGTC |
| *Il1b* | GAAATGCCACCTTTTGACAGTG | TGGATGCTCTCATCAGGACAG |
| *Tnf* | CTGAACTTCGGGGTGATCGG | GGCTTGTCACTCGAATTTTGAGA |
| *Gapdh* | CCCTTAAGAGGGATGCTGCC | TACGGCCAAATCCGTTCACA |
